# Fabrication and Performance Evaluation of Highly Sensitive Flexible Strain Sensors with Aligned Silver Nanowires

**DOI:** 10.3390/mi11020156

**Published:** 2020-01-30

**Authors:** Jae Hyuk Choi, Myung Gyu Shin, Young Jung, Dong Hwan Kim, Jong Soo Ko

**Affiliations:** 1 Graduate School of Mechanical Engineering, Pusan National University, Busandaehak-ro 63beon-gil, Geumjeong-gu, Busan 46241, Korea; wogur423@gmail.com (J.H.C.); young89@kitech.re.kr (Y.J.); akiocom55@gmail.com (D.H.K.); 2 Department of Electronics Engineering, Pusan National University, Busandaehak-ro 63beon-gil, Geumjeong-gu, Busan 46241, Korea; 3 Precision Manufacturing Control R&D Group, Korea Institute of Industrial Technology, 42-7, Baegyang-daero 804beon-gil, Sasang-gu, Busan 46938, Korea

**Keywords:** flexible strain sensor, silver nanowire, dip coating

## Abstract

In this study, we fabricated strain sensors by aligning silver nanowires and transferring them with polydimethylsiloxane (PDMS) and compared the performances of the fabricated strain sensors along the alignment direction. Two types of flexible strain sensors embedded with the aligned silver nanowires were fabricated: one in the longitudinal direction, which is the same as the alignment direction, and the other in the lateral direction, which is perpendicular to the alignment direction. We then evaluated their properties. The proposed longitudinally aligned strain sensor showed the maximum sensitivity (gauge factor (GF) = 89.99) under 25% tensile conditions, which is 7.08 times higher than the sensitivity (GF = 12.71) shown by the laterally aligned strain sensor under 25% tensile conditions. This finding confirmed that the alignment direction of silver nanowires influences the sensitivity of flexible strain sensors. Furthermore, this study demonstrates that the laterally aligned strain sensor (*ε* > 60%) can be used in wearable devices because it satisfies the required strain range (*ε* > 50%). Since the strain sensors were fabricated using the temperature-controlled dip coating process, they can be produced at low cost in large quantities, and thus they have advantages for commercialization. These characteristics will be applicable to various flexible devices as well as to flexible strain sensors.

## 1. Introduction

With the development of wearable devices, there has been active research on flexible and stretchable devices. Wearable devices are being used for various purposes, such as real-time monitoring of people’s movements, checking momentum and heart rate, and early diagnosis of diseases [1]. In particular, flexible strain sensors are being researched for their wide application in wearable devices, such as sensors attached to the skin for detecting vital signs and as sensors for detecting motions of large and small magnitudes in people [2]. Strain sensors detect mechanical force or object deformations and transform them into electric signals. There are several different types of strain sensors, including capacitive-type, resistive-type, liquid metal, piezoelectric, triboelectric, and so forth. However, it is difficult to apply some strain sensors such as liquid metal, piezoelectric and triboelectric type to practical applications of wearable devices due to their low resolution, poor dynamic performance and their sophisticated measurement [3]. However, capacitive-type and resistive-type sensors need only relatively simple measurement and offer high stretchability. For flexible strain sensors, resistive-type, and capacitive-type strain sensors are mainly used.

The conventional resistance-type strain sensors [4] and strain sensors based on semiconductor or microelectromechanical systems (MEMS) [5] generally have a low strain range (*ε* < 5%) and low sensitivity (GF < 2, GF = Gauge Factor). Furthermore, the strain sensors fabricated by conventional methods have limitations for body applications due to their low strain range (*ε* < 5%) and low biocompatibility [6,7]. Various flexible strain sensors are being developed to overcome these limitations and apply them to wearable devices.

Flexible strain sensors with a strain range (*ε* > 50%) applicable to wearable devices are generally fabricated as composites of polymers and nanomaterials. Selection of appropriate polymers and nanomaterials has been shown to provide reliable dynamic strain sensing. This combination enables the fabrication of high performance stretchable strain sensors using phenomena such as crack propagation and tunnel effects in conductive nanonetworks [3]. Nanomaterials such as graphene, carbon nanotubes (CNTs), and silver nanowires are frequently used to produce high-performance flexible strain sensors [8,9,10,11,12]. Flexible strain sensors fabricated using graphene have very high sensitivity (GF > 1500) due to the limitation of the two-dimensional material, but they have a low strain range (*ε* < 7%). Strain sensors fabricated using graphene with these characteristics are appropriate for applications requiring a low strain range and high sensitivity [8,9,10]. The CNT is one of the materials often used for the development of flexible strain sensors because it not only has strength that is several tens of times higher than that of iron, but it also has a high flexibility and conductivity [11]. Strain sensors fabricated by putting CNT fibers on a flexible substrate have a low sensitivity (GF < 1.5) over a broad strain range (*ε* < 200%). Furthermore, the strain sensor fabricated by placing CNT fibers after applying a pre-strain had a maximum strain range of 900%. The strain sensor showed sufficient performance as a flexible strain sensor for motion detection and other similar purposes, when only the strain range was considered (GF < 1.5, *ε* < 200%); however, low sensitivity, nonlinearity, and large hysteresis are the disadvantages that need to be improved [12].

Silver nanowire is a material that is expected to improve the low strain range of graphene and the low sensitivity of CNTs. Silver nanowires are frequently used in transparent electrodes, solar cells, and flexible devices based on excellent electrical, optical, and mechanical properties, and they are also used in flexible strain sensors [13,14,15,16,17,18,19]. Xu and Zhu (2012) produced silver nanowires embedded in the surface layer of PDMS by a method of coating a silicon wafer with silver nanowires, pouring liquid PDMS over it, curing it, and removing it. This method met the challenges of working with silver nanowires such as breakage of the contact part and permanent resistance change caused by short circuits. Xu and Zhu produced a capacitive strain sensor using this method, and it was the first study that fabricated a conductor by transferring silver nanowires to PDMS [17]. Amjadi et al. fabricated a strain sensor with a sandwich structure with PDMS covering silver nanowires at the top and bottom. For this strain sensor, randomly arranged silver nanowires were transferred to PDMS. A flexible strain sensor was then fabricated by pouring PDMS onto the silver nanowires exposed to the surface. The measurements of the sensor were more stable when the exposed silver nanowires were covered by PDMS. Furthermore, we fabricated a flexible strain sensor with a GF between 2 and 14 and a maximum strain range of 70% by adjusting the amount of silver nanowires. This result is more appropriate for application to wearable devices than other materials [18]. Kim et al. controlled the sensitivity and strain range of the flexible strain sensor by using the two variables, pre-strain and coating amount. The sensor was fabricated by randomly coating the silver nanowires using the vacuum filtration method and transferring the silver nanowires on the pre-stretched PDMS [19].

In this study, two types of strain sensors were fabricated using aligned silver nanowires: a longitudinally aligned strain sensor, in which the electrodes were connected in the alignment direction, and a laterally aligned strain sensor, in which the electrodes were connected perpendicular to the alignment direction. We evaluated the strain range and sensitivity of the fabricated sensors according to the alignment direction. In addition, we compared the performance of the strain sensor with randomly arranged silver nanowires with those of the strain sensors fabricated in our study and examined their differences. The silver nanowires were aligned using the temperature-controlled dip coating process, and the reliability of the fabrication process was assessed in terms of the degrees of alignment when the silver nanowires were arranged randomly, when they were aligned, and after the transfer.

## 2. Working Principle and Fabrication Procedure

### 2.1. Working Principle

**Figure 1:**
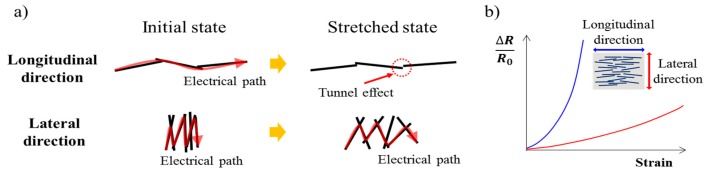
Working principle of the proposed strain sensor. (**a**) Working principle of the longitudinally and laterally aligned strain sensors. (**b**) Graph for predicting the resistance change rate according to the strain of the fabricated strain sensors.

The longitudinal strain sensor and the lateral strain sensor in this study had opposite characteristics. The resistance of the longitudinal strain sensor easily increases when strain is applied due to detaching nanowires. On the other hand, the resistance of the lateral strain sensor increased slowly when strain was applied due to the arrangement of the silver nanowires. As shown in Figure 1a, the silver nanowires in the lateral strain sensor were aligned perpendicular to the stretched direction. These arrangements caused rotation to the nanowires due to the PDMS shrinkage that occurred during tension. This phenomenon allows the lateral strain sensor to be highly stretchable.

### 2.2. Fabrication Procedure

**Figure 2:**
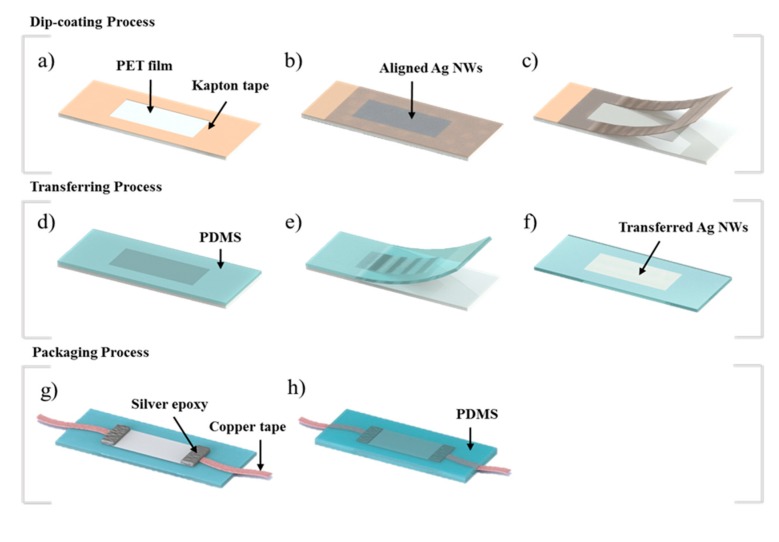
Total process diagram for the flexible strain sensor. (**a**) Forming a pattern on a polyethylene terephthalate (PET) film using Kapton tape. (**b**) Silver nanowire coating using the dip coating process on a patterned PET film. (**c**) Removal of the pattern used as a mask. (**d**) Curing after fabrication of polydimethylsiloxane (PDMS). (**e**) Transfer of the silver nanowires to the PDMS. (**f**) Flipped PDMS to which the silver nanowires were transferred. (**g**) Connecting wires to both ends of the PDMS to which silver nanowires were transferred. (**h**) Encapsulation of the sensor using the PDMS.

The fabrication process of the flexible strain sensor is largely composed of three steps. The first step is the dip coating process, which aligns the silver nanowires in one direction (Figure 2a–c). The second step is the transfer of the silver nanowires to PDMS (Figure 2d–f). In this process, the silver nanowires aligned on the substrate are transferred to the PDMS to embed the silver nanowires in the PDMS surface. The third step is packaging to connect the electrical wire to the PDMS embedded with silver nanowires to cover the surface connected by the electrical wire with PDMS (Figure 2g,h).

#### 2.2.1. Dip-Coating Process

**Figure 3:**
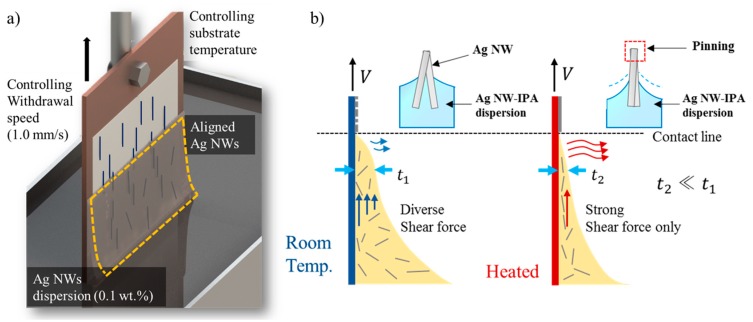
The principle of alignment of the silver nanowires. (**a**) Process schematic. (**b**) Principle of the alignment of silver nanowires in the temperature-controlled dip coating process.

The dip coating process aligns silver nanowires (DT-AGNW-N30, Dittotechnology, Gyeonggi-do, Korea) on a polyethylene terephthalate film (PET film, ES301425, Good fellow, UK), which was used as the substrate. In this study, we used the temperature-controlled dip coating process. When the temperature is controlled, the same degree of alignment can be obtained at 1.0 mm/s, which is 10 times faster than when the temperature is not controlled [20,21]. Figure 3 shows the principle of the temperature controlled dip coating process. As the solution receives a shear force from the substrate that is pulled up, and the nanowires are aligned by the shear force caused by the relative movement of the substrate and the solution pulled up by the substrate [22]. When the solution thickness is decreased by adjusting the solution temperature and the coating speed, the shear force is received by the nanowires in the solution, which facilitates the alignment [21]. In this study, a temperature of 60 °C and a speed of 1.0 mm/s were used as the process conditions. The dip coating process was performed 50 times per sample. The test equipment was composed of a dip coating device (Auto Dipcoater, DAO TECHNOLOGY, Gyeonggido, Korea), power supply (DP 30-03TP, TOYOTECH, CA, USA), hot plate (PC 620D, CORNING, Corning, NY, USA), stainless steel container, and heating plate. The heating plate was fabricated using aluminum plates with good thermal conductivity and strength (FCK 42, MONO, Seoul, Korea) and an electric plate to generate heat (CF-HF5V10W, CAMPREE, Seoul, Korea). The heating plate was designed in a sandwich structure with the electric plate in the middle covered by two aluminum plates.

**Figure 4:**
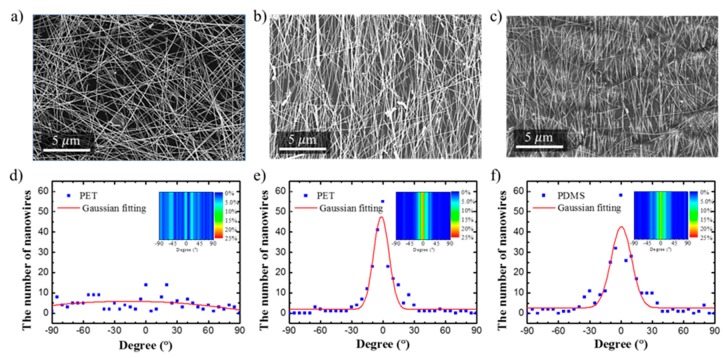
Comparison of the degree of alignment of silver nanowires. (**a**) SEM image of unaligned silver nanowires. (**b**) SEM image of the silver nanowires aligned using the temperature-controlled dip coating process. (**c**) SEM image of the silver nanowires transferred to the PDMS. (**d**) Analysis of the degree of alignment of unaligned silver nanowires. (**e**) Analysis of the degree of alignment of silver nanowires aligned using the temperature-controlled dip coating process. (**f**) Analysis of the degree of alignment of the transferred silver nanowires. Insets of (**d**–**f**) show the distribution of nanowires according to the angle, and the amount of silver nanowires is expressed in color.

Figure 4b shows the result of the dip coating process. The degree of alignment was analyzed using SEM images. To evaluate the degree of alignment of the silver nanowires aligned by the dip coating process, the silver nanowires were randomly coated and compared. The PET film coated by random silver nanowires was fabricated using the drop-casting process. The angle of the silver nanowires (θ) was manually measured through the SEM image using the Image J program. The baseline when measuring the angle was based on the direction in which the substrate is pulled up during the dip coating process, which was set at 0°. For bent silver wires, the angle of the line interconnecting the two end points of the silver nanowires was measured.

#### 2.2.2. Transferring Process

In this process, the transfer technique was used to the transfer silver nanowires coated on the PET film to the PDMS. In general, polymer and silver nanowires do not stick well together [4]. However, when the transfer technique was used, the silver nanowires were buried in the PDMS. The silver nanowires between the cured PDMS and the transferred surface become a composite of PDMS and silver nanowires [17]. The PDMS (Sylgard 184 kit, Dow corning, Midland, MI, USA) was prepared by mixing the principal matter and the hardener at a weight ratio of 10:1. The air bubbles were removed from the mixed PDMS using a vacuum desiccator (VDR-20, JEIO TECH, Daejeon, Korea). Then the silver nanowires were transferred to the PDMS with a thickness of 500 μm. The PDMS was cured in a vacuum oven (OV-2, JEIO TECH, Korea) at 70 °C for 2 h. Figure 4c shows the SEM image of the PDMS embedded with silver nanowires after the transfer process was completed.

Figure 4a shows the SEM image of silver nanowires that were randomly coated on the PET film. Figure 4b shows the SEM image of silver nanowires aligned on the PET film by the dip coating process. Figure 4c shows the SEM image of silver nanowires transferred to the PDMS. Figure 4d–f show the degree of alignment of silver nanowires in each SEM image verified using a full width half maximum (FWHM) expression. Figure 4d shows a graph analyzing the degree of alignment of silver nanowires that were randomly coated in Figure 4a. Figure 4e shows a graph for analyzing the degree of alignment of silver nanowires aligned using the dip coating process on the PET in Figure 4b. Figure 4f shows a graph for analyzing the degree of alignment of silver nanowires transferred to the PDMS in Figure 4c. In this study, FWHM was used to express the degree of alignment of silver nanowires. A lower FWHM value indicates a higher degree of alignment. The high degree of alignment allows the longitudinal strain sensor to have high sensitivity since the contact of nanowires is easily detached by strain. On the other hand, the lateral strain sensor had lower sensitivity because the contact was maintained for a longer time even when tension was applied. The FWHM value shown in the Figure 4f (after transferring) was 23.0135°, somewhat larger than the FWHM value shown in the Figure 4e (before transferring) of 17.3734°. This means that after the transfer, the degree of alignment of silver nanowires decreased. The reason for this reduction could be found in the process of transferring the silver nanowires to the PDMS. The PDMS transfer process is a process of removing the cured PDMS after its permeation between the nanonetworks formed by the silver nanowires. In the dip coating process, the initially coated silver nanowire layer was attached to the PET by strong van der Waals forces, which made it difficult to transfer to the PDMS. For this reason, the degree of alignment of the silver nanowires transferred to the PDMS decreased.

#### 2.2.3. Packaging Process

The first step in the packaging process is to connect the electric wires. The wires were connected to the surface of the PDMS embedded with silver nanowires. On the surface of the PDMS in which silver nanowires were embedded, some of the silver nanowires are exposed to the surface, which enables electrical connection. A silver nanowire solution (1.0 wt %) was drop-casted at the position on the surface of the PDMS embedded with silver nanowires where the electrical wire would be connected. The adhesive part was reinforced by adding one conductive layer between the conductive adhesive and the silver nanowires transferred to the PDMS. After that, the wire connection part was electrically connected more strongly using silver epoxy (CW-2400, Chemtronics, Kennesaw, GA, USA), which has good polymer adhesion. The wires were fabricated using a Cu tape (OPM, Seoul, Korea). The exposed wire connections and silver nanowires exposed to the surface were encapsulated by casting PDMS to a thickness of 500 μm. The exposed wire connections and silver nanowires exposed to the surface were encapsulated using PDMS over the fabricated samples.

## 3. Results and Discussion

**Figure 5:**
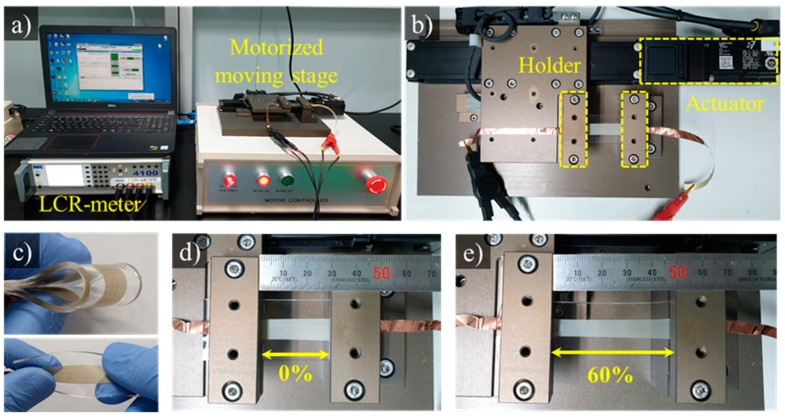
Configuration of test equipment. (**a**) Tensile test machine. (**b**) Motorized moving stage. (**c**) Fabricated sensors. (**d**) Fabricated sensors fixed to the motorized moving stage. (**e**) Stretching the sensor by 60%.

Figure 5 shows the test equipment we used to evaluate the performance of the proposed flexible strain sensors. The tensile test machine was composed of a linear motor (LX2001CP, Misumi, Japan) and a linear axis encoder (SRSH24YN-200, Misumi, Japan). The initial value, tensile distance, and tensile speed can be adjusted using the Lab View application. Electric signals were acquired using an LCR meter (LCR 4110, Wayne Kerr Electronics, London, UK) and two wires. In this study, we mainly measured the resistance changes. The fabricated sensors were measured in real time by connecting the LCR meter with a computer (Figure 5).

**Figure 6:**
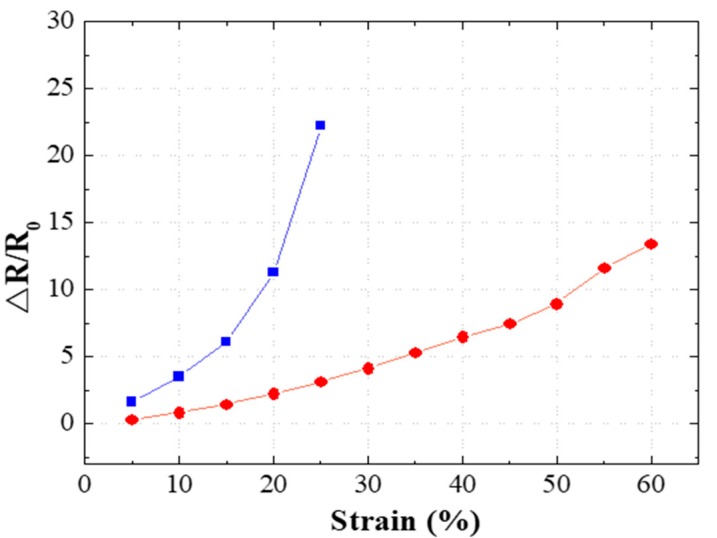
Graph of the resistance change rate of the longitudinally and laterally aligned flexible strain sensors.

To compare the fabricated flexible strain sensors, the changes in the resistance of the sensors were measured according to the range of the applied strain, which are presented in Figure 6. Loading/unloading experiments were repeated in each strain range with the fabricated strain sensors. For the presented data, we used a mean value of 15 resistance measurements after the strain sensors were stabilized in the corresponding strain range. The longitudinally aligned strain sensors were stretched by up to 25%, and the laterally aligned strain sensors were stretched by up to 60%. For the longitudinally aligned strain sensors, GF = 89.99 was measured with a 25% stretch. For the laterally aligned strain sensor, GF = 12.71 and GF = 22.10 were measured with 25% and the 60% stretch, respectively. The sensitivity of the strain sensor was measured by GF, which is defined as $\left(\Delta R/R_{0}\right)/\left(\Delta L/L_{0}\right)$. ΔR denotes the resistance change, $R_{0}$ denotes the initial resistance, ΔL denotes the length change, and $L_{0}$ denotes the initial length. The measurement results of the longitudinally aligned strain sensor show that disconnection occurred easily at the contact point of silver nanowires and the sensitivity was high even in a low strain range sensor because strain was applied in the alignment direction of silver nanowires. The laterally aligned strain sensor was subject to strain that was perpendicular to the silver nanowire alignment direction. As a result, the electrical connection between the silver nanowires at the contact point was maintained for a longer period. Hence, GF did not increase much even in a relatively high strain (60%) condition.

**Figure 7:**
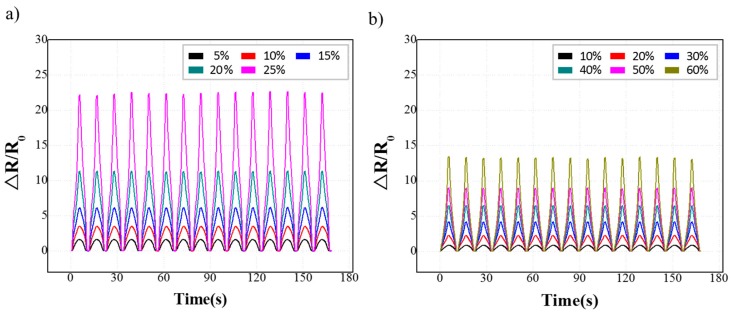
Loading/unloading behaviors. (**a**) Measurement of resistance change rate according to the elongation of the longitudinally aligned flexible strain sensor. (**b**) Measurement of resistance change rate according to the elongation of the laterally aligned flexible strain sensor.

Figure 7 shows how resistance changes occur evenly by repeatedly applying strain to the fabricated sensors. Figure 7a shows the measurements of resistance changes according to the strain in the longitudinally aligned strain sensor. We performed 15 repeated experiments over time. To effectively compare the resistance change rate in each strain range, the time it takes for one repetition was set to 10 s. For the longitudinally aligned strain sensor, an increase in the maximum strain range from 5% to 25% increased the sensitivity $\left(\Delta R/R_{0}\right)$ from 1.62 to 22.50. For the laterally aligned strain sensor, when the maximum strain range increased from 10% to 60%, the sensitivity $\left(\Delta R/R_{0}\right)$ increased from 0.85 to 13.09. This result was consistent with the prediction that the longitudinally aligned strain sensor has a relatively narrow strain range and high sensitivity, while the laterally aligned strain sensor has low sensitivity and a relatively broad strain range. Furthermore, the values measured in each cycle were also stable.

**Figure 8:**
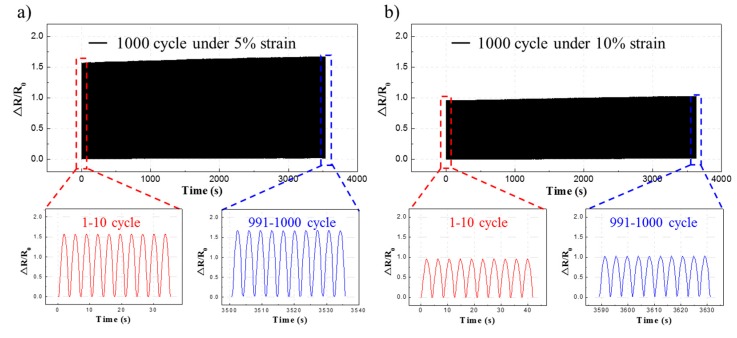
Repeated loading/unloading tests. (**a**) Result of repeated tensile tests of the longitudinally aligned flexible strain sensor. (**b**) Result of repeated tensile tests of the laterally aligned flexible strain sensor.

Figure 8 shows a graph for the durability test of the fabricated sensors. For each sensor, 1000 cycles of repeated loading/unloading tests were performed. Each sensor was tested in the strain range corresponding to 1/5 of the maximum strain range for stable measurement. The longitudinally aligned strain sensors were subjected to a repeated strain of 5% while the laterally aligned strain sensors were subjected to a repeated strain of 10%. For the longitudinally aligned strain sensor, the value of $\Delta R/R_{0}$ under a 5% strain in the 1000th cycle was higher than the value of the first cycle by 6.07%. For the laterally aligned strain sensor, the value of $\Delta R/R_{0}$ under a 10% strain in the 1000th cycle was higher by approximately 6.49% than the value of the first cycle. The resistance change rate increased because the contact of the nanowires became unstable due to the sliding effect and buckling phenomenon resulting from the repeated strain [18]. The flexible strain sensors fabricated in this study formed a thinner silver nanowire layer than those of the previous studies [18,19]. As a result, the resistance in the repeated tensile tests increased somewhat.

**Figure 9:**
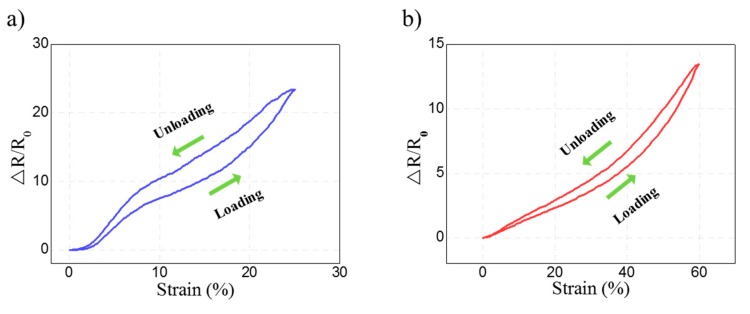
Hysteresis of strain sensors. (**a**) Hysteresis of the longitudinal strain sensor. (**b**) Hysteresis of the lateral strain sensor.

**Figure 10:**
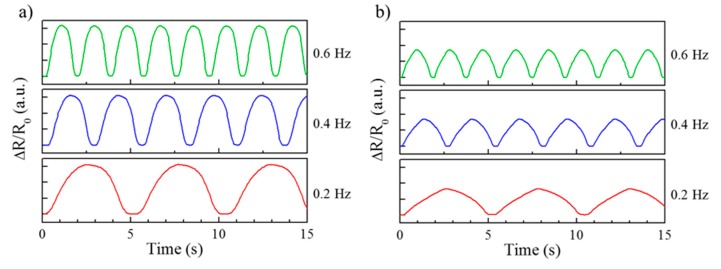
Frequency experiment. (**a**) Measurement of the resistance change rate according to the frequency of the longitudinally aligned flexible strain sensor at 5% elongation. (**b**) Measurement of the resistance change rate according to the frequency of the laterally aligned flexible strain sensor at 10% elongation.

Figure 9 shows a graph for the hysteresis of the fabricated sensors. Figure 9a illustrates the hysteresis curve for the longitudinal strain sensor. As shown in the figure, the relative change in the resistance does not overlap with that in the loading–unloading cycle for the longitudinal strain sensor. On the other hand, Figure 9b shows that the hysteresis performance of the lateral strain sensor was more stable than the longitudinal strain sensor. The flexible strain sensors are attached to detect motions on the body parts that cause repeated rapid changes, such as arms, knees, and wrists. Therefore, whether the values are measured evenly with no significant changes under different frequency conditions in flexible strain sensors is an important factor [23]. Figure 10 shows the result of evaluating flexible strain sensors fabricated in different frequency conditions. In the case of the longitudinally aligned strain sensors, the measured value of $\Delta R/R_{0}$ was 1.5475 at 0.2 Hz, 1.5600 at 0.4 Hz, and 1.5774 at 0.6 Hz. When the increase in resistance was verified using these sensors, the resistance increased by approximately 1.93% at 0.6 Hz. In the case of the laterally aligned strain sensor, the measured value of $\Delta R/R_{0}$ was 0.8245 at 0.2 Hz, 0.8514 at 0.4 Hz, and 0.8592 at 0.6 Hz. Thus, the resistance increased by approximately 4.21% at 0.6 Hz. Even though the waveforms were uniform, the resistance change rates for both types of sensors increased as the frequency increased.

**Figure 11:**
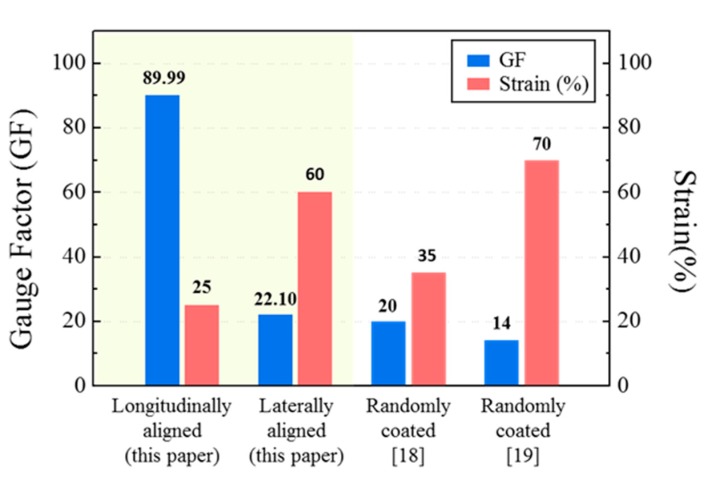
Graph comparing the gauge factor (GF) and strain ranges of the longitudinally and laterally aligned strain sensors between this study and previous studies.

Figure 11 shows a graph comparing the strain sensor with randomly arranged silver nanowires that were researched previously and the two types of strain sensors fabricated in this study. In the longitudinally aligned strain sensor, the maximum GF measurement was 89.99 in the 25% strain range. This value is more than five times higher than the flexible strain sensor researched using the same material in the same strain range and under the same strain condition (*ε* = 25%) [19]. This result shows that the longitudinally aligned strain sensor proposed in this study can measure the small motions that are difficult to be detected by existing strain sensors fabricated by randomly arranging the silver nanowires. Furthermore, the maximum GF of the laterally aligned strain sensor was measured at 22.10 in the range of up to 60%. This had a lower strain range but a higher sensitivity compared to those presented in previous studies [18]. The nanonetwork of laterally aligned strain sensors was stable, and the sensitivity was lower than that of the longitudinally aligned strain sensor, but it was higher than that of existing strain sensors. This can be attributed to the large number of silver nanowires used in coating existing strain sensors by the drop casting process; this process on the other hand, coated the silver nanowires thinly (1.5 μm) and uniformly using the dip coating process.

## 4. Conclusions

In the present study, instead of fabricating strain sensors by randomly arranging silver nanowires, we fabricated two types of flexible strain sensors (longitudinal and lateral) embedded with aligned silver nanowires and compared and analyzed their properties and performances. The longitudinally aligned strain sensor demonstrated a narrow strain range (*ε* < 25%), but the GF, which indicates sensitivity, was high at 89.99, which is more than five times higher than that of a previous study with the same strain conditions (*ε* = 25%) [19] and 7.08 times higher than that of the laterally aligned strain sensor. In the laterally aligned strain sensor, sensitivity (GF < 22.10) was relatively low, but the strain range (*ε* < 60%) was high. These results confirmed that the alignment direction affects the sensitivity and strain range of flexible strain sensors embedded with silver nanowires. Furthermore, the results of our experiments confirmed uniform signals in a strain range of up to 25% for longitudinally aligned strain sensors and in a strain range of up to 60% for laterally aligned strain sensors. The durability evaluation was performed for 1000 cycles at 5% elongation for the longitudinally aligned strain sensor and at 10% elongation for the laterally aligned strain sensor, with a frequency from 0.2 to 0.6 Hz. This study verified the characteristics of strain sensors according to the alignment direction of silver nanowires embedded in PDMS. It is expected that these strain sensors will be applicable to various flexible devices as well as to flexible strain sensors developed using this principle.
